# Prone-position thoracoscopic resection of posterior mediastinal lymph node metastasis from rectal cancer

**DOI:** 10.1186/s12957-015-0466-0

**Published:** 2015-02-12

**Authors:** Yasuhiro Shirakawa, Kazuhiro Noma, Takeshi Koujima, Naoaki Maeda, Shunsuke Tanabe, Toshiaki Ohara, Toshiyoshi Fujiwara

**Affiliations:** Department of Gastroenterological Surgery, Dentistry and Pharmaceutical Sciences, Okayama University Graduate School of Medicine, 2-5-1 Shikata-cho, Kita-ku, Okayama, 700-8558 Japan

**Keywords:** Thoracoscopic resection, Prone position, Mediastinal lymph node metastasis, Colorectal cancer

## Abstract

Mediastinal lymph node metastasis from colorectal cancer is rare, and barely any reports have described resection of this pathology. We report herein a successful thoracoscopic resection of mediastinal lymph node metastasis in a prone position. A 65-year-old man presented with posterior mediastinal lymph node metastasis after resection of the primary rectal cancer and metachronous hepatic metastasis. Metastatic lymph nodes were resected completely using thoracoscopic surgery in the prone position, which provided advantages of minimal invasiveness, good surgical field, and reduced ergonomic burden on the surgeon. Thoracoscopic resection in the prone position was thought to have the potential to become the standard procedure of posterior mediastinal tumors.

## Background

Colorectal cancer most commonly metastasizes to the regional lymph nodes, liver, lungs, brain, and bone [1,2]. Mediastinal lymph node metastasis from colorectal cancer has been reported on rare occasions [3-5]. With the continued development of chemotherapeutic options, the control of metastatic colorectal cancer has increased in recent years. Furthermore, some good results from aggressive surgical resection of distant metastasis or local recurrence have been reported [6-9]. The general consensus is that solitary metastatic lesions from colorectal cancer should be resected. However, very few reports have described the surgical treatment of mediastinal lymph node metastasis, because of the rarity of this pathology. We present a case of mediastinal lymph node metastases successfully treated using thoracoscopic resection in the prone position.

## Case presentation

A 65-year-old man with stage IIIa rectal cancer underwent low anterior resection and lymph node dissection in January 2009. Histopathological findings showed only one vicinal lymph node metastasis. After the first surgery, he received adjuvant chemotherapy comprising uracil-tegafur for 1 year, followed by 10 cycles of 5-florouracil, leukovorin and oxaliplatin (FOLFOX).

Twenty-six months after the first surgery, liver metastasis was detected on computed tomography and ^18^ F-fluorodeoxyglucose (^18^ F-FDG) positron emission tomography (PET). Serum level of carcinoembryonic antigen (CEA) was also high (30.48 ng/ml). A solitary liver mass measuring 3 × 2 × 2 cm in lateral segment 2 was identified, and lateral segmentectomy was performed. The pathological finding was metastatic adenocarcinoma, similar to the primary rectal cancer. After the second surgery, he could not receive adjuvant chemotherapy because of the adverse drug reaction.

**Figure 1:**
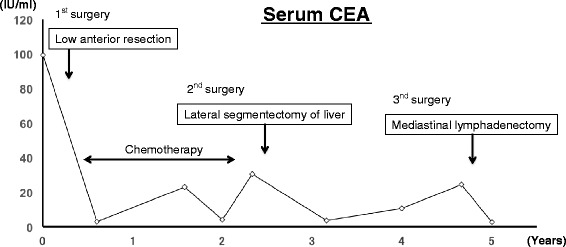
**Treatment progress and changes in serum CEA levels.**

**Figure 2:**
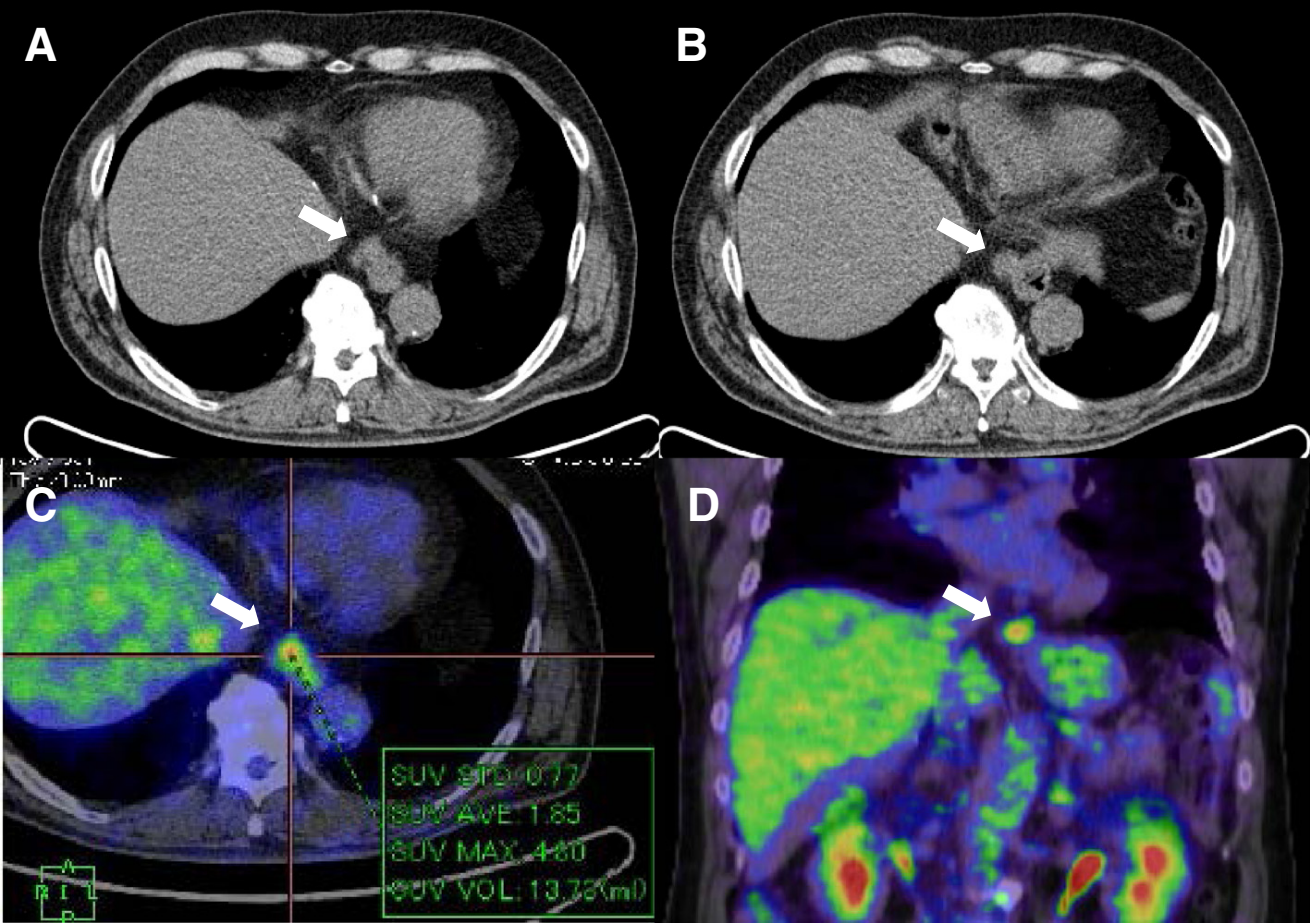
**CT and PET-CT findings of posterior mediastinal lymph nodes. (A, B)** CT shows multiple enlarged lymph nodes (arrows). **(C, D)** PET-CT shows significant accumulation (SUVmax, 4.80) in the posterior inferior mediastinum (arrows).

At 29 months after this second surgery, serum CEA level was again found to be high (24.33 ng/ml) (Figure 1). CT revealed a lower posterior mediastinal mass close to the lower esophagus (Figure 2A, B). One month later, whole body PET-CT showed FDG accumulation in the mass (Figure 2C, D). No other sites of FDG accumulation were detected. Solitary posterior mediastinal lymph node metastasis from rectal cancer was diagnosed and the decision was made to resect the lesion by thoracoscopic surgery in the prone position.

**Figure 3:**
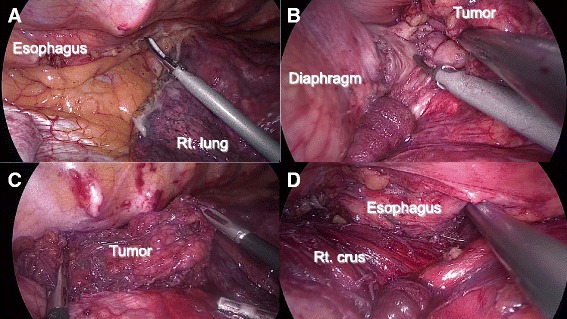
**Surgical procedure by thoracoscopic surgery in the prone position. (A)** Dissection of the right lower pulmonary ligament. **(B)** Separation of metastatic lymph nodes from diaphragm and esophagus. **(C)** En bloc resection of lymph nodes. **(D)** The right crus and esophagus are recognized after resection of lymph nodes.

**Figure 4:**
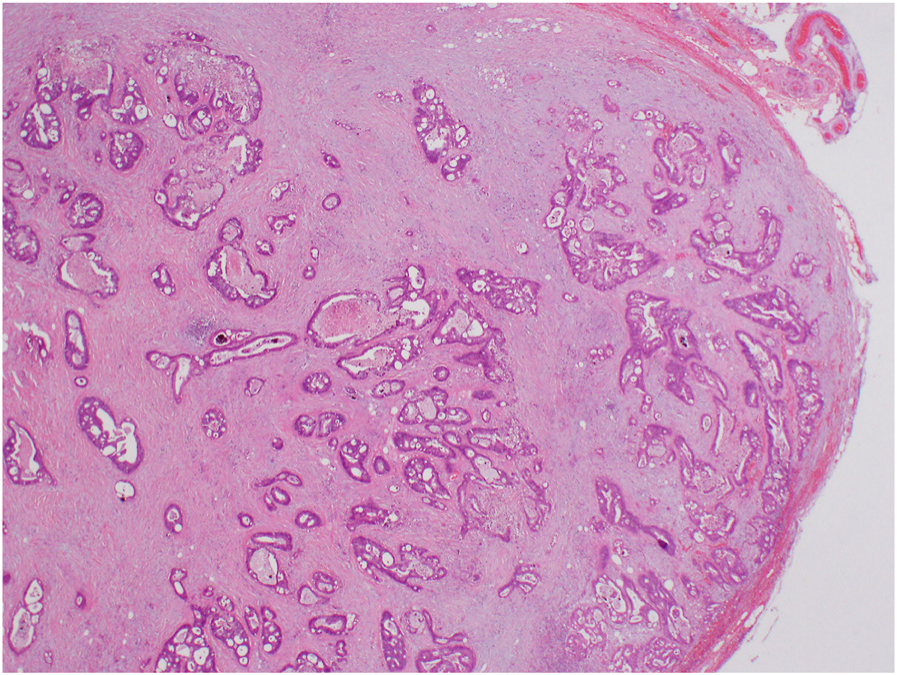
**Histopathological examination of the resected specimen shows metastatic lymph nodes involving adenocarcinoma.**

Thoracoscopic resection was performed. The procedure was as follows. First, a 12-mm port for the operator’s right hand was inserted into the fifth intercostal space on the posterior axillary line with 8 mmHg of pneumothorax. Second, a 12-mm camera port was inserted into the ninth intercostal space on the lower scapula line. Third, a 5-mm port for the operator’s left hand was inserted into the seventh intercostal space on the posterior axillary line. Finally, a 12-mm port for the assistant using cotton swabs was inserted into the eighth intercostal space on the middle axillary line. A mass involving multiple lymph nodes was visualized between the esophagus and esophageal hiatus and was separated from the esophagus using an ultrasonic coagulation and cutting device. No tumor invasion or adhesions around the tissue were identified, and successful en bloc resection was achieved (Figure 3). Histopathological examination showed metastatic lymph nodes containing adenocarcinoma similar to the primary rectal cancer and liver metastasis (Figure 4). Postoperatively, serum CEA level again decreased (Figure 1). The patient was administered gastric stasis and anticholinergic drugs because of slight postoperative gastric stasis.

Recently, the development of diagnostic imaging modalities such as PET-CT and adjuvant chemotherapy has changed the therapeutic plan for patients with metastatic and recurrent colorectal cancer, and some good results for distant metastasis or regional recurrence have been reported with aggressive resection. However, few reports have described the resection of solitary mediastinal lymph node metastasis from colorectal cancer [7-10]. Standardized therapeutic approaches have also yet to be defined. The differential diagnoses for mediastinal lymphadenopathy are tuberculosis, sarcoidosis/sarcoid-like reaction, lymphoma, and metastatic lymph nodes [11-13]. The usefulness of PET-CT for identifying metastatic mediastinal lymph nodes has been reported. However, PET-CT is useless for tuberculosis, sarcoidosis, and lymphoma because of the low specificity [14]. This modality is also useless for non-small cell lung cancer (NSCLC) because of the low sensitivity [15]. In the present case, we diagnosed solitary posterior mediastinal lymph node metastasis from rectal cancer because of FDG accumulation and increasing serum CEA levels. The postulated mechanism leading to mediastinal lymph node metastasis is spread via lymphatic drainage routes from liver metastasis or via the paravertebral and para-aortic lymphatic plexus in patients with abdominal or pelvic malignancy. In the present case, we speculated the spread involved lymphatic drainage from liver metastasis because of the previous liver metastasis.

The key advantage of thoracoscopic surgery compared with thoracotomy is the marked reduction in invasiveness. We have reported the usefulness of the prone position for thoracoscopic esophagectomy [16]. In this case, we applied the technique to resect posterior mediastinal lymph nodes. The prone position provided a good view of the surgical field without displacing the heart or right lung using gravity and artificial pneumothorax. Moreover, cotton swabs assisted in reducing the ergonomic burden on the surgeon around the esophageal hiatus. These advantages of thoracoscopic surgery with the prone position were thought to contribute to the good postoperative course and have potential to become standard procedures in the treatment of posterior mediastinal tumors.

## Conclusions

We have described successful thoracoscopic resection of mediastinal lymph node metastasis from rectal cancer. Thoracoscopic surgery in the prone position has the potential to become the first-choice surgical approach for posterior mediastinal tumors because of the advantages in terms of minimum invasiveness, good surgical field, and reduced ergonomic burden for the surgeon.

## Consent

Written informed consent was obtained from the patient for publication of this case report and the accompanying images.
